# Better agonist for the opioid receptors

**DOI:** 10.1186/s13065-018-0383-8

**Published:** 2018-02-08

**Authors:** Syed Lal Badshah, Asad Ullah, Salim S. Al-showiman, Yahia Nasser Mabkhot

**Affiliations:** 1 Department of Chemistry, Islamia College University, Peshawar, 25120 Pakistan; 2 Department of Chemistry, College of Sciences, King Saud University, Riyadh, Saudi Arabia

**Keywords:** Opioid receptors, Analgesics, Agonists, Molecular docking, Selectivity

## Abstract

This commentary highlights the recent work published in journal Nature on the structural based discovery of novel analgesic compounds for opioid receptors with minimal effects. Manglik et al. selectively targeted the Gi based μOR pathway instead of the β-arrestin pathway of the opioids. The computational screening of millions of compounds showed a list of several competent ligands. From these ligands they synthesized the compounds with the best docking score, which were further optimized by adding side residues for better interaction with the μOR. A promising compound, PZM21, was a selective agonist of μOR. It has better analgesic properties with minimal side effects of respiratory depression and constipation. This work is a step towards better drug designing and synthesizing in terms of efficacy, specificity with least side effects of targeted GPCR proteins present in the human proteome.

## Introduction

Morphine is the natural alkaloid present in opium and it is obtained from poppy plant. Opium has been used as an analgesic and as a recreational drug since ancient times. Other common analgesics used include natural alkaloids like codeine, oxycodone, etc. where addiction and other side effects are an increasingly apparent social problem. Current progress in the discovery of different opioid receptors has helped the search for receptor specific drugs without adverse side effects. The protein data bank now contains high resolution structures of the μ, δ, к and nociception opioids receptor proteins [1–5]. The opioid receptors are G-protein coupled receptors (GPCRs), whose signaling is mediated through the G proteins [1]. In the last few years, there has been a surge in high resolution X-ray crystallographic structures of GPCRs; particularly from the Kobilka research group at Stanford University. Whose work resulted in the Nobel Prize of Physiology in 2012 [6, 7].

The GPCR proteins are important players in eukaryotic signaling mechanisms [8, 9]. They transfer the message from extracellular side to the intracellular side of the cell across the plasma membrane [8, 9]. The common ligands for GPCRs includes lipids, fatty acids, neurotransmitters, photons, cytokines, hormones and metal ions [8, 9]. They transduce the signal across the plasma membrane by binding with these ligands that causes certain conformational changes into the seven trans-membrane alpha helices of GPCRs [8, 9]. The GPCR proteins are important drug targets and it is estimated that around 30% or more of the available marketed drugs are for GPCR related diseases [9]. There is a general consensus that around 350 GPCRs are involved in various human diseases. Another ~ 100 GPCRs (called orphan GPCRs) have little information available about their natural ligands or physiological function [10]. In the last few years several structures of GPCRs were computationally explored through molecular docking approaches to find suitable agonist and antagonist compounds that have no adverse effects [9, 11, 12]. Similarly, those GPCR whose X-ray crystallographic structures are not available were studied using the homology modeling techniques, where suitable ligands were docked with them based on virtual screening methods [9, 12].

In a recent study, Manglik et al. search for ideal opioid ligands that have lower side effects [13]. They took around three million compounds from the ZINC database library [14, 15] and docked them with the orthosteric site of the μOR [13]. Each compound has more than a million different configurations in the binding site that were considered. Most of the ligands interacted with the Aspartate147 of the orthosteric site of the protein [13]. The top 2500 ligands were evaluated for their novelty and interaction with various internal residues. The new ligands selected have binding affinities in the micromolar (μM) range. These newly predicted ligands are cationic amines that mostly bind with μOR and show unique interactions of hydrogen bonding with Asp147, which was not reported before in the literature [13]. For better binding affinity and selectivity, new analogues of these ligands were made. They retained the parent compound interaction with the receptor; however the additional side groups made new interactions in the binding site. The analogues that make several interactions in the molecular docking studies were synthesized in the laboratory for further studies [13].

**Fig. 1:**
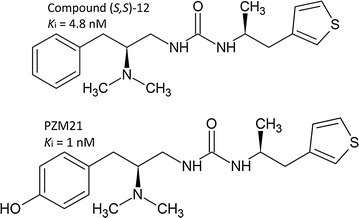
Chemical structure of compound (*S, S*)-12 and PZM21 [13]

From the series of synthesized stereoisomeric compounds, compound 12 (Fig. 1), expressed better binding affinity with μOR and resulted in the specific activation of G_i/o_ and very low initiation of β-arrestin-2 pathway [13]. To increase interaction in the binding pocket, they introduced a hydroxyl group in this compound at the *para* position on the benzene ring (Fig. 1). The new potent synthetic (*S, S*)-21 compound is named as PZM21 [13]. It makes nine interactions within the allosteric site with the μOR and more favorable binding free energy. The G_i/o_ activation assay showed an EC_50_ value of 4.6 nM and 76% efficacy [13]. The analgesic efficiency of PZM21 is higher than that of morphine [13]. The PZM21 is a highly selective agonist of μOR while it has no agonist activity for other opioid receptors or neurotransmitter transporters [13]. The PZM21 possess the concentration dependent analgesic effects in a mouse hotplate assay [13]. Its metabolism in mice liver is quite slow, and 8% of the drug is metabolized in 1 h. Its analgesic time in mice is 180 min which is longer than both morphine and TRV 130 [13]. In her news and views published in Nature, Brigitte Kieffer showed the comparison between PZM21 and TRV130 [16]. The TRV130 is specific pain relieving analgesic that has lower side effects, similar to PZM21 [16]. The TRV 130 drug is currently in the third phase of its clinical trials though it also has some side effects like respiratory depression [13]. Experiments on mice showed that PZM21 provided relief in pain for which the response is mediated through the CNS only while responses mediated through that of the spinal nerves are ignored [16]. Further experiments on mice showed that PZM21 did not result in addiction, making it a more suitable analgesic as compared to other available drugs [13]. Further studies are required to determine the metabolic stability and pharmacokinetics of PZM21 and its derivatives [13]. PZM21 can be synthesized from the method presented by Manglik et al. from (*S*)-amino acid amides and thiophene-3-carbaldehyde in few steps or it may be synthesized through other simple routes and can be easily commercialized [13].

Now with more powerful femtosecond serial X-ray crystallography, a number of high resolution crystal structures of GPCRs are available [17–19]. In the next few years we likely will have hundreds of high resolution structures of GPCRs from all of its different classes. Computational molecular docking approaches with highly selective agonists and antagonists will be available for each protein that will have minimal side effects [20]. In crystal form, most GPCRs have an inactive state and there are always ambiguities in the interaction of agonist/antagonist with the protein. Molecular dynamics simulations should always be performed to get a more flexible and active state of GPCRs. The main advantages of structural based optimization and selection of ligands are that it saves both time and money in order to choose the best ligand for specific GPCR that work only through a single sided pathway in a biological system.
